# Microbiome 2.0: lessons from the 2024 Gut Microbiota for Health World Summit

**DOI:** 10.1080/19490976.2024.2400579

**Published:** 2024-09-10

**Authors:** Sushrut Jangi, Gail Hecht

**Affiliations:** a Department of Medicine, Tufts Medical Center, Boston, MA, USA; b Department of Medicine, Loyola University Chicago, Maywood, IL, USA

**Keywords:** Fecal microbial transplantation, probiotics, inflammatory bowel disease

## Abstract

This Meeting Summary highlights the key insights from the 12th meeting of the Gut Microbiota for Health World Summit, held in Washington, DC, organized by the American Gastroenterological Association (AGA) and the European Society of Neurogastroenterology and Motility (ESNM). Through a 2-day series of plenary sessions, workshops, a poster session, and live discussions involving thought leaders, physicians, researchers, and representatives from the Food and Drug Administration and the pharmaceutical industry, the conference attendees focused on the strategies and challenges in developing microbiome-based therapies to prevent and treat human disease. The conference highlighted progress in the field, including the recently successful introduction of 2 new fecal microbial transplantation-based products into the clinical setting, and the continuing development of next-generation probiotics. However, to continue to advance microbiome-directed treatments, three key themes emerged during the meeting, including (1) better methods to identify actionable targets in the microbiome (2) developing effective strategies to manipulate the microbiome (3) aligning microbiome-based therapies with existing treatment paradigms in the real world.

In March 2024, the American Gastroenterological Association (AGA) and the European Society of Neurogastroenterology and Motility (ESNM) organized the 12th meeting of the Gut Microbiota for Health World Summit in Washington, DC. This 2-day conference brought together gastroenterologists, basic scientists, microbiologists, immunologists, nutritionists, and representatives from the Food and Drug Administration and the pharmaceutical industry. The planned focus of the meeting was on the potential for manipulation of the gut microbiome in health and disease. The meeting included four plenary sessions on (1) improving health and disease using the microbiome (2) bringing new microbiome-based products to market (3) moving from human-derived microbiome therapies toward synthetic communities (4) how to better handle big data and the microbiome. The meeting also included four workshops, focused on correcting microbial functional deficits, identifying and using biomarkers in the microbiome, personalizing nutrition to change the microbiome, and the future of microbiome therapies. A poster session showcased several research abstracts and offered an opportunity for participants to network and ask questions. Every plenary session and workshop was followed by an open question-and-answer period which provided for lively and engaged audience feedback and response. These plenary sessions, workshops, speakers, panelists, and attendees underlined that the next wave of microbiome research should move beyond correlative microbiome studies toward developing generalized treatment strategies that can effectively modify the microbiome to maintain health, prevent the development of human illness and improve the outcomes of established diseases. In this summary, we chose to highlight the strategies and challenges surrounding three key themes that repeatedly emerged during the meeting that can move us closer toward successfully developing microbiome-based therapies (Figure 1), including (1) identifying actionable targets in the microbiome (2) developing strategies to effectively manipulate the microbiome (3) aligning microbiome-based therapies with existing treatment paradigms in the real-world. The studies cited in this summary were either referenced and discussed during presentations, the question-and-answer period, poster sessions, or in personal communications with attendees.

**Figure 1.**
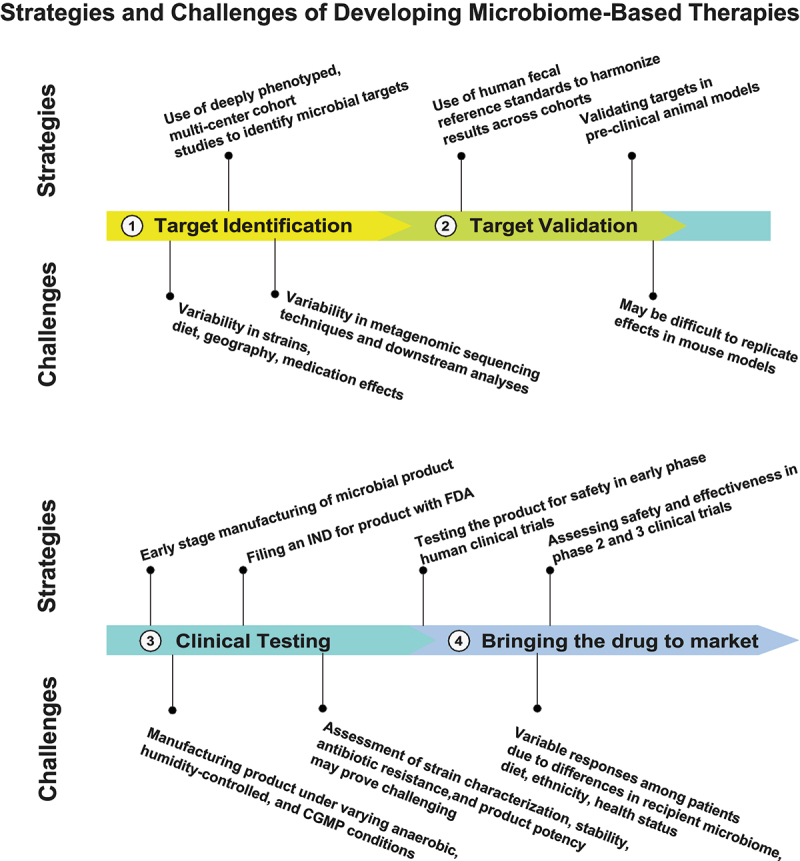
Developing microbiome-based therapies: strategies and challenges.

Four phages are illustrated in developing a microbiome-directed product, including identifying a microbial target, validating the microbial target, determining a therapeutic strategy to modulate the identified target, and leveraging these findings toward bringing a drug to market. The strategies (above each bar) and challenges (below each bar) are given for each phase.

## Identifying microbial targets in the microbiome

### Roadblocks to characterizing microbial targets

Developing microbiome-based therapies requires identifying a disease-specific target: either a microbe that contributes to disease pathogenesis (a pathobiont) or a beneficial microbe that prevents or ameliorates disease. Comparing the microbiomes of diseased patients with healthy individuals may reveal such targets. However, identifying highly reproducible microbiome signatures specific to disease states has remained challenging. Partly, this may be due to complex confounders influencing the cohort microbiome, including host-specific factors (diet, genetics, medication exposure) and study-specific factors (cohort design, location of study center, and sample collection methodology). Furthermore, variability in metagenomic sequencing techniques across research centers can introduce bias at nearly every step, including in the choice of DNA extraction kits, storage techniques, read processing, and statistical analysis.^1^ In the MOSAIC Standards Challenge, in which 44 laboratories submitted results of 7 shared reference standards to assess the impact of methodological design of microbiome studies, choosing whether to use 16s sequencing vs shotgun metagenomic sequencing substantially influenced downstream analyses.^1^ While 16s sequencing showed an elevated Firmicutes to Bacteroidetes ratio, shotgun metagenomics underlined the inverse result, raising concerns about the comparability of results between the two sequencing strategies.

### Proposed strategies to improve target identification

#### Deeper characterization of patient cohorts

Despite the above challenges, host-microbiome studies remain foundational for the identification of therapeutic microbial targets. Careful cohort design may help to surmount the effect of potential confounders. Choosing patients across distinct geographical sites may allow for differentiation of true disease-driving species from disease-unrelated passenger strains.^2^ Pairing traditional self-reported data such as food-frequency questionnaires with the measurement of dietary biomarkers from serum or stool within a subset of the cohort may more accurately capture dietary intake within the cohort.^3^ Collection of app-based patient-reported outcomes, continuous metabolic monitoring via subcutaneous sensors, and anthropometric data can allow for deeper characterization of patient phenotypes.^2^ The use of preclinical prospective cohort designs, such as the GEM study surveying the microbiome of first-degree relatives of inflammatory bowel disease (IBD) patients, may identify microbes that drive early subclinical pathogenesis, potentially solving the “chicken and egg problem” that confounds whether differentially abundant microbes are the cause or consequence of the illness.^4^ Collecting samples from interventional studies may allow for the inclusion of better controls and greater insight into treatment-effects and underlying mechanism. Finally, the use of artificial intelligence models geared toward microbiome inputs, such as Microbiome Differentiable Interpretable Temporal Rule Engine (MDITRE), may better leverage longitudinal, time-series data to reveal microbial targets associated with host disease phenotypes.^5^

#### Replicability and validity of microbial targets

Replicability has been a major challenge in microbiome research.^6^ While the use of reporting guidelines and consistent approaches to sequencing and analysis pipelines can improve cohort replicability,^6^ the availability of a Human Fecal Reference Material, soon to be introduced commercially by the National Institute of Standards and Technology, may facilitate meaningful comparisons of sequencing results across research centers.^1^ This Human Fecal Reference Material was prepared by collecting stool samples from healthy donors, followed by homogenization and storage at −80°C, with its subsequent detailed characterization including metagenomic sequencing, flow cytometry, culturomics, and metabolomics.^7^ In future microbiome studies, labs can order and use this reference standard to benchmark their protocols and characterize biases in their pipelines.

After a disease-specific microbial target has been identified within a cohort, the microbe’s effects can be tested in a preclinical animal model. Such an approach has been utilized to validate the pathogenic effects of clinical IBD-associated bacterial strains of *Klebsiella pneumoniae* in a colitis-prone germ-free mouse model.^2^ Similarly, an *Akkermansia muciniphila* strain identified in lean humans could reverse high-fat diet-induced metabolic disorders, including insulin resistance, in an obesity mouse model.^8^ Through a combination of large data-mining across patient cohorts, subsequent validation in appropriate preclinical mouse models can generalize an approach for identifying microbiome therapeutic cores for each disease.

## Effective manipulation of the microbiome

### Roadblocks to manipulating the microbiome

Once pathogenic or beneficial microbial targets are identified, strategies to effectively eliminate pathobionts or introduce favorable microbes into the human gut remain challenging. Targeting pathobionts with antibiotics, for example, is problematic as such therapies can have multiple off-target effects, leading to the overall disruption of the microbiome or acquisition of resistance. On the other hand, introducing beneficial microbes via fecal microbial transplantation (FMT) has variable efficacy for treating conditions outside of *Clostridiodes difficile* associated disease, with unmeasurable components in the FMT product, an inability to easily tailor FMT to the individual, and the potential to introduce deleterious microbes into the recipient. Using probiotics as a vehicle to introduce beneficial strains into the host can also be challenging, as identified strains may fail to engraft, and may not meaningfully alter downstream functional pathways or health outcomes. Novel treatment strategies beyond antibiotics, FMT, and conventional probiotics may help surmount these roadblocks. Developing novel microbiome-directed treatments, however, also requires overcoming regulatory hurdles, including developing an Investigational New Drug application with the Food and Drug Administration (FDA).

### Proposed strategies to improve microbial manipulation

#### Targeting pathobionts

Eliminating disease-causing microbes in humans without impacting the surrounding microbiome could be accomplished using ecologically-directed treatment approaches. Bacteriophages are a promising tool to manipulate bacteria, as these viruses can infect bacteria with high specificity without harming the host.^9^ In a proof-of-concept study, Elinav and colleagues showed that a 5-phage cocktail therapeutic could specifically eradicate pathogenic strains of *Klebsiella pneumoniae* and ameliorate colonic inflammation in germ-free IBD-prone mice. Their work provides a framework to identify phage combinations that can target a disease-causing pathobiont, confirm the efficacy of the treatment in a mouse model, and bring the resulting phage therapy into trials for human disease.^2^ Alternative approaches engineering phages or bacteria to carry CRISPR-Cas9 systems, or utilizing postbiotics, or microbially-produced molecules such as bacteriocins, could selectively eliminate the pathobiont.

#### Incorporating beneficial microbes into the gut microbiome

##### Next generation probiotics

To move beyond the limitations of conventional probiotics or FMT, newer approaches include the development of next-generation probiotics, or human-centric microbial strains, that may prevent or treat illness. Everard and colleagues demonstrated that a beneficial strain of *Akkermansia muciniphila* identified in human cohorts and tested in animal models, could be scaled up for interventional trials. Administration of this strain was safe and well-tolerated, improved insulin sensitivity, total cholesterol, and reduced body weight and liver dysfunction in a cohort of 32 overweight and obese volunteers.^10^ Similar approaches have led to ongoing clinical trials testing *Faecalibacterium prausnitzii* in IBD. Challenges inherent to developing next-generation probiotics include difficulties in culturing and manufacturing specific strains at industrial scale and engrafting them into the host successfully. Efforts to improve strain-selection, effective use of pasteurization, and incorporating nutritional therapies to improve engraftment, may help to optimize outcomes of these therapies.

##### Simple defined microbial consortia

Single-strain probiotics may have limited effect, as it may be difficult for a single strain to integrate into the complex microbial community and render a therapeutic impact. In contrast, simple defined microbial consortia, consisting of 8–12 strains, can potentially overcome such challenges. VE303, a consortium of 8 commensal *Clostridia* strains, reduced the rate of recurrent *Clostridioides difficile* infection compared to placebo.^11^ Similarly, GUT-108, composed of 11 bacterial strains, could reduce a broad range of inflammatory cytokines (IL-1B, IFN-γ, TNF-α) and could reverse colitis in a T-cell mediated mouse model.^12^ Other microbial consortia are being tested to improve the outcomes of cancer immunotherapy, and to prevent allergic diseases in newborns. Despite the promises of simple defined consortia, these microbial groups may still lack the diversity and abundance to effectively engraft into the host microbiome. Integrating more scaffold strains – or bacteria that help support the therapeutic core of a consortium – may be necessary for the consortia to take root. Furthermore, simple consortia may possess far fewer metabolic pathways than may be required to induce a metabolic change in the host.

##### Complex defined microbial consortia

Complex consortia, with more than 100 strains of microbes, could provide the scaffolds and functional potential to meaningfully alter the recipient microbiome. Cheng and colleagues developed Hcom2, a complex consortium of more than 119 of the most abundant gut microbes that provide for 62 different functional pathways, which could be sustainably engrafted into mice after 4 weeks.^13^ In a proof-of-concept study, they demonstrated that such consortia could be highly engineerable, with a plasmid introduced into a *Bacteroides* strain able to increase secondary bile acid production and reduce fibrosis in a nonalcoholic fatty liver disease model. However, the logistical challenges of purifying each strain from human-derived fecal samples, scaling up production, and testing the efficacy of complex consortia in human trials remains a significant hurdle.

#### Surmounting regulatory challenges

Developing an FMT-based product or a next-generation probiotic requires an Investigational New Drug (IND) Application with the FDA. An IND generally requires an assessment of the microbial strains within the product, antibiotic resistance profiles, the genomic sequence of the relevant strains, details of the manufacturing process, stability data, and potency testing. Once a research group or company is ready to move the product into clinical trials, they can request a pre-IND meeting with the FDA to discuss ways to best prepare the IND. The success of this regulatory pathway has already been demonstrated by the recent FDA’s approval of microbiome-based products such as Vowst and Rebyota.

## Incorporating microbiome therapies into personalized, real-world treatment paradigms

### Roadblocks to treating patients with microbial therapies

While there has been proof-of-concept success in identifying disease-associated microbes and the subsequent development of microbiome-directed treatments, whether such therapies can provide meaningful clinical benefits with real-world applications remains a challenge. Though the most full-spectrum microbiome-directed therapy to date – FMT – has been shown to increase recipient bacterial diversity and supplant pathogenic microbial populations with beneficial microbes, the clinical benefits in complex disease has remained variable and unpredictable.^14^ In a meta-analysis of 6 randomized controlled trials evaluating the role of FMT in obesity and metabolic syndrome, there was no difference in obesity parameters, with negligible improvements in hemoglobin A1c, insulin sensitivity, and metabolic profiles.^15^ Even in ulcerative colitis, FMT has had mixed success, with a trial randomizing active ulcerative colitis patients to a stool-based product (SER-287) unable to achieve pre-specified remission outcomes compared to placebo, leading to termination in the development of this product.^16^ While microbiome-directed therapies beyond FMT are gaining increasing interest for treating complex diseases, several factors complicate the success of these treatments, including (1) how to personalize therapeutic plans to an individual’s microbiome (2) the complementary role of diet and (3) the effective positioning of these treatments alongside available therapies and in the context of disease phase.

### Proposed strategies to optimize the efficacy of microbiome-directed therapies

#### Personalizing microbiome-directed treatments

Incorporating high-resolution, longitudinal microbiome sequencing studies into multi-center clinical trials of microbiome-directed products, may provide more actionable insights into how recipient profiles may better predict clinically meaningful endpoints of the microbiome-directed treatment.^17^ Customized machine-learning modeling methods may also better predict donor-recipient complementarity.

Personalizing treatments toward correcting functional rather than taxonomic deficits may also prove to be a more clinically meaningful target. Technologies that can rapidly screen individuals for alterations in major gut-derived metabolites, such as short chain fatty acids (SCFAs), secondary bile acids, or aryl hydrocarbon receptor (AhR) agonists, could help to select personalized microbial treatments that redirect production of the missing metabolite.^18^ For example, as some patients with IBD are known to have reduced levels of stool Ahr agonists, treatment with next-generation probiotics exhibiting Ahr-agonist production capacity, such as *Lactobacillus reuteri*, may allow for a more clinically impactful match of therapy to individual microbial profiles, as has been demonstrated in using *L. reuteri* to correct the metabolic syndrome.^19^

#### Aligning microbiome-based therapies with patient diet

Diet can skew the host microbiome toward specific compositions. Those who eat a diet composed mostly of whole plant foods, for example, are likely to have increased delivery of fermentable fibers to the distal colon, resulting in increased populations of potentially beneficial SCFA-producing bacteria.^20^ Randomized controlled trials of fiber interventions predictably alter microbiome composition or activity, showing promise for personalized diets, prebiotics, probiotics, and synbiotics in engineering the microbiome.^21^ While trials of healthy controlled diets have been shown to reduce weight, improve insulin sensitivity, and increase life expectancy,^20,22^ whether diets can influence the efficacy of microbial-based therapies is unclear. In one trial, pairing FMT with an anti-inflammatory diet was better able to sustain remission in ulcerative colitis patients over 1 year, compared to standard medical therapy.^23^ However, whether specific diets support the engraftment of microbially-directed therapies in humans remains largely unknown. The recent Precision Nutrition 2020–2030 NIH Strategic Plan for nutrition research may provide foundational evidence for diet-microbiota interactions, informing personalized diet design that could improve the efficacy of microbiome-based therapies.^24^

#### Positioning microbiome-directed therapies in current treatment paradigms

The timing of how and when to deploy microbiome-based therapies alongside conventional therapies is largely unknown. The phase of the illness may impact the outcome of the treatment. Immunosuppressive therapies such as tumor necrosis factor inhibitors given to patients with IBD, for instance, are known to improve intestinal dysbiosis, suggesting that a colon in remission may be more likely to support the growth of healthy bacteria. Utilizing this concept, treating the host using conventional immunosuppressives, then utilizing microbiome-based therapies to *maintain* a healthy disease state may prove to be a useful paradigm, although studies illustrating this concept have largely not been performed.

## Next steps

**Figure 2.**
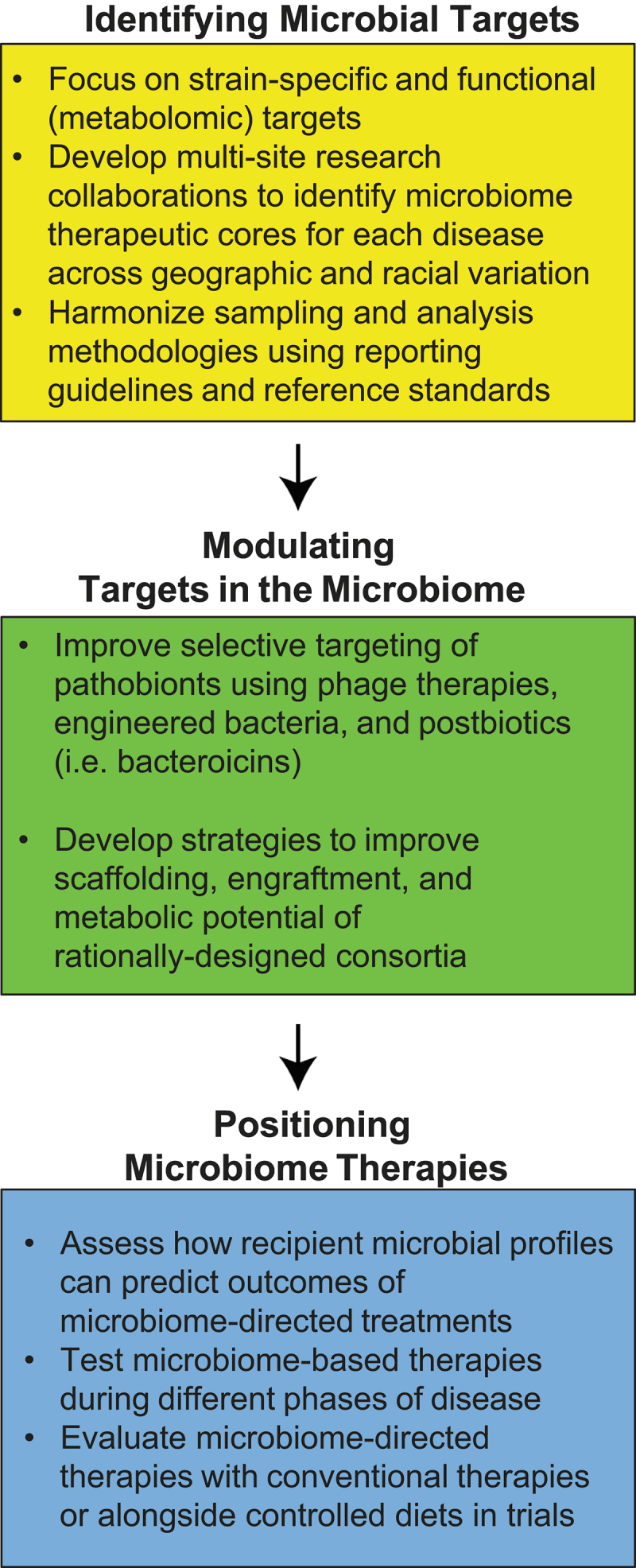
Action items to improve development of microbiome-directed therapies.

The 2024 Gut Microbiota for Health World Summit highlighted that there has been progress in microbiome-based interventions for improving human health. In the last few years, 2 new FMT-based products have been approved by the FDA and are being given to treat recurrent *Clostridiodes difficile* associated disease. Countless next-generation probiotics are being developed and tested to improve outcomes in chronic human diseases, while novel approaches to developing consortia are extending the promise of tailored treatment options. Rapid growth in sequencing technologies, downstream analyses, and predictive modeling are powering a new wave of data-driven microbiome studies, informing the development of more generalized microbiome-based treatments. However, knowledge gaps to advance these therapies remain (Figure 2). Future studies centered on these gaps may help to better achieve clinically meaningful impacts of microbiome-directed therapies, providing novel, potentially safe and efficacious options for patients with otherwise difficult to manage chronic diseases.

Knowledge gaps and proposed action items are suggested over 3 thematic areas: identifying microbial targets, modulating identified targets, and positioning microbiome-directed therapies.
